# A variant in the 5′UTR of *ERBB4* is associated with lifespan in Golden Retrievers

**DOI:** 10.1007/s11357-023-00968-2

**Published:** 2023-10-19

**Authors:** Robert B. Rebhun, Daniel York, Flora M. D. De Graaf, Paula Yoon, Kevin L. Batcher, Madison E. Luker, Stephanie Ryan, Jamie Peyton, Michael S. Kent, Joshua A. Stern, Danika L. Bannasch

**Affiliations:** 1 Department of Surgical and Radiological Sciences, University of California, Davis, CA USA; 2 Department of Population Health and Reproduction, University of California, Davis, CA USA; 3 Veterinary Medical Teaching Hospital, University of California, Davis, CA USA; 4 Department of Medicine and Epidemiology, University of California, Davis, CA USA

**Keywords:** Dog, Canine, Cancer, Longevity, Aging, *ERBB4*, *HER4*, 5’UTR, GWAS, Golden Retriever, Geroscience, Lifespan

## Abstract

Genome-wide association studies (GWAS) in long-lived human populations have led to identification of variants associated with Alzheimer’s disease and cardiovascular disease, the latter being the most common cause of mortality in people worldwide. In contrast, naturally occurring cancer represents the leading cause of death in pet dogs, and specific breeds like the Golden Retriever (GR) carry up to a 65% cancer-related death rate. We hypothesized that GWAS of long-lived GRs might lead to the identification of genetic variants capable of modifying longevity within this cancer-predisposed breed. A GWAS was performed comparing GR dogs ≥ 14 years to dogs dying prior to age 12 which revealed a significant association to *ERBB4*, the only member of the epidermal growth factor receptor family capable of serving as both a tumor suppressor gene and an oncogene. No coding variants were identified, however, distinct haplotypes in the 5′UTR were associated with reduced lifespan in two separate populations of GR dogs. When all GR dogs were analyzed together (*n* = 304), the presence of haplotype 3 was associated with shorter survival (11.8 years vs. 12.8 years, *p* = 0.024). GRs homozygous for haplotype 3 had the shortest survival, and GRs homozygous for haplotype 1 had the longest survival (11.6 years vs. 13.5 years, *p* = 0.0008). Sub-analyses revealed that the difference in lifespan for GRs carrying at least 1 copy of haplotype 3 was specific to female dogs (*p* = 0.009), whereas survival remained significantly different in both male and female GRs homozygous for haplotype 1 or haplotype 3 (*p* = 0.026 and *p* = 0.009, respectively). Taken together, these findings implicate a potential role for *ERBB4* in GR longevity and provide evidence that within-breed canine lifespan studies could serve as a mechanism to identify favorable or disease-modifying variants important to the axis of aging and cancer.

**Supplementary Information:**

The online version contains supplementary material available at 10.1007/s11357-023-00968-2.

## Introduction

There is increased interest in studying aging in companion dogs as a model for human aging because companion dogs share the same environment with humans and display similar causes of mortality such as cancer, neurodegenerative diseases, and heart disease [1]. In addition, companion dogs are subject to naturally occurring diseases, and have access to medical care including vaccination, preventative medicine, diagnostics, and treatment of otherwise life-limiting diseases. Furthermore, the common practice of euthanasia for perceived poor quality of life in dogs with debilitating diseases of aging favors the dog as a model of healthy aging [2, 3]. With proper study design, companion dogs can also be evaluated for potential effects of hormonal and environmental exposures, diet, obesity, exercise, and genetics [3, 4]. Finally, studies of aging in companion dogs allow for titration in the design of studies to evaluate more (or less) inbred populations by examining at-risk breeds or studying aging using within or across-breed studies [5–7].

Human candidate gene studies have identified variants that are reproducibly associated with specific diseases or longevity [8]. For example, APOE isoforms are known to be associated with cardiovascular disease, Alzheimer’s disease, stroke, and diabetes [9], which comprise four of the top seven leading causes of death in people. Genome-wide association studies (GWAS) have further confirmed associations with apolipoprotein E (APOE) when comparing extreme longevity individuals to younger controls [10–12]. To the authors’ knowledge, however, APOE has not been specifically implicated in cancer. Interestingly, disease-specific studies investigating human extreme longevity found that centenarians do not necessarily lack common complex disease risk alleles, but rather show a greater delay in the onset or progression of major diseases [13], consistent with the concept of geroscience [14]. Taken together, these prior findings point to the possibility that protective genetic factors may exist and could play an important role in extreme longevity [15]. This could be explained by the existence of favorable gene variants or by the “buffering” mechanism proposed by Bergman and others, where unfavorable genes are buffered or tempered by favorable alleles in other genes [16, 17]. Although the majority of human and canine genetic studies are designed to search for genes associated with disease, an alternative “positive biology” approach shifts the focus to identifying genes associated with desirable phenotypes such as longevity or healthy aging [18].

In contrast to the human population where cancer accounts for only 21% of deaths within the USA, the Golden Retriever (GR) breed carries up to a 65% cancer-associated mortality rate, with the most common tumor types being hemangiosarcoma (23%), lymphoid neoplasia (18%), other sarcomas (16%), and carcinomas (13%) [19, 20]. Complex trait mapping studies in dogs have demonstrated the power and utility of GWAS, resulting in within-breed analyses requiring far less individuals than in similar human studies [21–23]. GWAS studies have successfully identified many genes associated with phenotypic differences or genetically linked diseases in dogs [24–29] including some genes potentially related to cancer susceptibility [30–33]. Several GWAS studies in GR dogs have sought to identify possible genes associated with the incidence of common cancers in this breed [31–36], and although candidate variants have been found to be associated with the diagnosis of common cancers in GRs, none have specifically examined or accounted for lifespan. With such a high cancer-associated mortality rate, it is possible that one or more cancer predisposition genes could be fixed within the GR breed, and therefore within-breed GWAS may not be ideally suited for identifying a causative variant or variants. In contrast, a “positive biology” approach aimed at identifying genetic variants that favor longevity may consequently identify variants that counteract cancer risk or progression in this cancer-predisposed population of dogs. Based on the premise of favorable genes, we hypothesized that studies comparing long-lived GRs could be used to identify genes associated with longevity in this cancer-predisposed population.

## Methods

### Phenotype and sample collection

The archived DNA database within the UC Davis Center for Companion Animal Health was searched for blood samples collected from GRs presenting at the UC Davis Veterinary Medical Teaching Hospital (VMTH). Samples from GR mixes were excluded. Date of birth (DOB) and date of death (DOD) were extracted from the electronic medical record. If DOB or DOD were not available, follow-up phone calls or email communications with the primary care veterinarians or owners were made to document DOB or DOD. If only the year was known for DOB, then June 15 of that year was assigned as the DOB. For outside submissions, blood samples were collected at primary veterinary practices and submitted by the owner along with DOB. DOD for outside submissions was ascertained by follow-up phone or email communications with the owners or the primary care veterinarian. Signed owner consent was received at the time of hospital admission to the VMTH or at the time of submission for outside samples (UC Davis IACUC #18561, and #22865). DNA was extracted from 1 to 2 mL of whole blood using the Qiagen Puregene Blood Kit according to the manufacturer’s instructions.

### Genome-wide association study

All samples were genotyped using the Illumina CanineHD 220 k BeadChip (Illumina, San Diego, CA, USA). PLINK v1.9 software [37] was used for quality control and pruning the data set for minor allele frequency below 5% and individuals and single-nucleotide variants (SNVs) with more than 10% missing genotypes, leaving 140,343 SNVs in the analysis. Association analysis was performed using PLINK v1.9. The GR population was checked for the presence of outliers by means of a multidimensional scaling (MDS) analysis using PLINK v1.9. A scatterplot of the first two dimensions of the MDS coordinates was used to identify outliers. A QQ plot was used to check genomic inflation. A Bonferroni correction was applied to correct for 140,343 independent tests, resulting in a *p*-value of 3.56 × 10^−7^ as a genome wide significance threshold. All plots were generated in R v3.4.1 (Rstudio team, 2016) using GENABEL and qqman libraries [38, 39]. The top SNV was checked for LD with other SNVs in the region using R GENABEL software. A linear mixed model including an estimated kinship matrix as a covariate was used to control genomic inflation using GEMMA 0.97 software [40].

### Whole-genome sequencing

To identify genetic variants and their probable effect within the genomic region surrounding the top SNP identified by GWAS, the following pipeline was used. Whole-genome sequencing (WGS) of 8 GR was performed using Illumina HiSeq4000 platform at the DNA Technologies and Expression Analysis Core Laboratory, University of California Davis Genome Center. Four of the GRs were homozygous for the top identified SNP (chr37: 19,560,543; A/A) and had a lifespan that exceeded 14 years of age, and four dogs were homozygous for the alternate allele (G/G) and died prior to 12 years of age. The WGS data was aligned to Canfam 3.1 reference genome [41]. Based on *r*^2^ LD of the top SNP on chr37 and visualizing genes in the region in UCSC genome browser, variants from the region chr37:18000042–20145745 were extracted for further analysis. Variants were called using Samtools [42] and were filtered for inclusion with a MAF above 5%. The VCF files were then transformed into a TPED and TFAM file using PLINKv1.9. An association analysis was run on all the variants comparing the 4 long-lived GRs to the 4 normal life expectancy GRs. UCSC variant annotation integrator (VAI) was used for variant effect prediction. NCBI RefSeq genes, curated and predicted, of the CanFam3.1 Sept. 2011 assembly were used as a reference. Variants that were fully segregating with the phenotypes were pulled out from the VAI to provide the final analysis files. Since not every INDEL was called with the VAI tool, the uncalled INDELs were visually inspected to predict their effect and position in a gene using UCSC genome browser [43]. Canfam3.1, NCBI RefSeq, and Other RefSeq were used as tracks.

### Sanger sequencing

Primers used to amplify the *ERBB4* 5′UTR region were designed by Primer 3 (Forward: 5′-CCAGCTTCATTTTCTGCAAG-3′; Reverse: 5′-GACTGGAGGTGCAAGGAAAC-3′). PCR amplification was carried out using Kapa Long Range PCR kit (Roche, Wilmington, MA) in 12.5 µL reactions with 1.5 mM MgCl and 5% DMSO. Quality of PCR amplicons was analyzed with agarose gel electrophoresis. Unincorporated primers and dNTPs were removed from PCR products using ExoSAP-IT (USB, Cleveland, OH). The PCR amplicons were sequenced using BigDye Terminator v. 3.1 Cycle Sequencing Kit on an ABI 3500 Genetic Analyzer and analyzed using Sequencher v. 5.1 software (Gene Codes Corp, Ann Arbor,MI).

### Fragment analysis

Primers used to amplify the *ERBB4* 5′UTR region for fragment analysis were designed by Primer 3 (Forward: 5′ FAM-AAATGGCATCTCCCCTGTC-3′; Reverse: 5′-CAACCAGTGCGAGAAAGTGA-3′), with the 5′ forward primer labeled with FAM dye. PCR amplification was carried out similarly as above in 25 µL reactions. Fragment analysis was performed on an ABI 3500 Genetic Analyzer with GeneScan 400HD ROX dye size standards (Applied Biosystems) and analyzed using GeneMapper 4.1 (Applied Biosystems).

### Statistics

Statistical methods for the GWAS are included above. For the remaining analyses, we used Excel (Microsoft) and Prism software (GraphPad Software Inc.) for graph generation and statistical analysis. Kaplan–Meier curves were generated and median survival times were estimated. Dates of death (event), including euthanasia, were known for all dogs and no subjects were censored. To compare survival outcomes between subgroups, the log-rank (Mantel Cox) test was used for multiple group comparisons and the Gehan-Breslow-Wilcoxon test for comparing two groups. *P* < 0.05 was considered statistically significant.

## Results

### Genome-wide association study

**Fig. 1:**
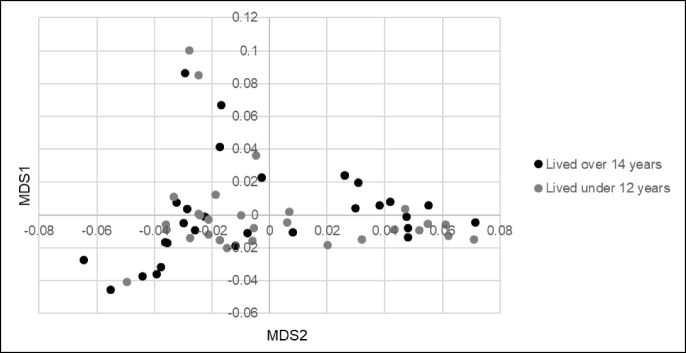
Multidimensional scaling plot of the Golden Retrievers used in the study. Dimensions one (*y*-axis) and two (*x*-axis) are plotted against each other. As made visible, the cases and controls are mixed evenly throughout the MDS plot

**Fig. 2:**
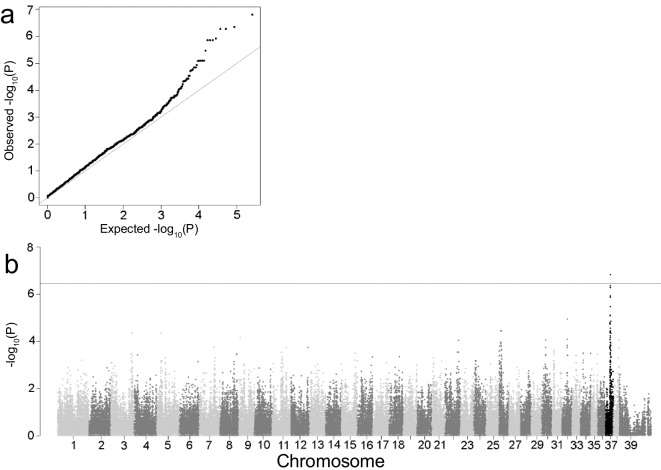
**a** Quantile–quantile plot, demonstrating observed versus expected *p*-values. λ = 1.2157. **b** Manhattan plot of longevity association in Golden Retrievers. The chromosome with the top SNV is highlighted in black. SNVs above the horizontal line meet Bonferroni significance criteria (*p*-value of 3.56 × 10^−^.^7^)

**Fig. 3:**
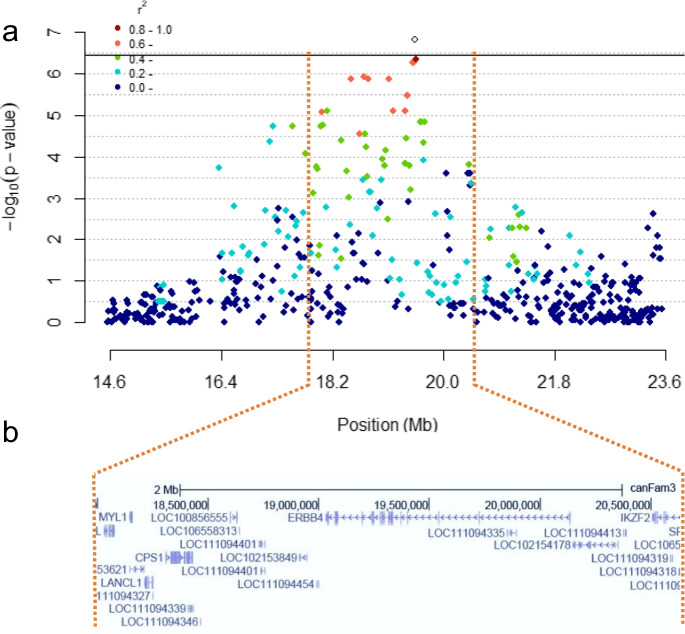
**a** Detailed view of the highest associated SNV on chromosome 37 (marked as a white diamond), plotted by base pair on the *x*-axis and *p*-value on the *y*-axis. SNVs are color coded based on *r*^2^ value to show extent of linkage disequilibrium. Horizontal bar represents Bonferroni significance line (*p*-value of 3.56 × 10^−^.^7^). **b** Genes in high LD region of top SNP (UCSC browser, CanFam 3.1, refseq predictor track)

To identify age associated loci, 58 GRs were genotyped using the Illumina CanineHD 220 k BeadChip. A case–control GWAS analysis was performed with 29 GRs who died before 12 years of age versus 29 GRs who lived longer than 14 years of age. Demographics of these 58 GRs are presented in supplemental Table 1. After filtering, 140,343 SNVs remained in the analysis. The MDS plot of the first two dimensions (Fig. 1) showed equal clustering of the cases and controls, indicating no significant outliers. The QQ-plot showed some genomic inflation (lambda 1.2) (Fig. 2a). A single genome wide significant locus was identified on chromosome 37 with a *p*-value of 1.52 × 10^−7^ (Fig. 2b). The top SNV is BICF2P171822, located at chr37:19,560,543, with genotype A/A in dogs above 14 years. A close-up of the region showing linkage disequilibrium (LD) with the highest associated SNV is shown in Fig. 3.

### Whole-genome sequencing

Using Samtools Variant calling, 9276 variants (6501 SNPs and 2775 indels) were identified within the associated region chr37.18000042–20145745. After segregation filtering in the 4 long-lived cases and 4 controls, 250 variants remained, including 25 INDELs and 225 SNPs. UCSC VAI showed that no variants were predicted to cause protein coding changes. Eleven annotations that were coded as INDELS did not get picked up by the variant effect predictor and were visually analyzed in UCSC genome browser to expose their effect. These 11 INDELS were intronic or intergenic. More details on the location of the variants can be found in Table 1.

**Table 1:** Detailed description of predicted effect of the variants segregating with the longevity phenotype, found after WGS in 4 case and 4 control dogs

	Total segregating	In ERBB4
Intron	162	156
Intergenic	77	0
Upstream	118	0
Downstream	3	0
Other	0	0

No missense or other protein coding changes were found (other). Variants that were present in ERBB4 are listed in column 2

Since no protein coding variants were identified, visual inspection of the region encompassing the *ERBB4* open reading frame (ORF, chr37:20,134,256–20,135,092) for structural variants that segregated between the 8 dogs was performed. This region included sequence upstream of the 5′ untranslated region (5′UTR) of exon 1 through part of the first intron. The only structural variants identified were insertion/deletions in the 5′UTR.

### Characterization of 5′UTR variants in ERBB4

**Fig. 4:**
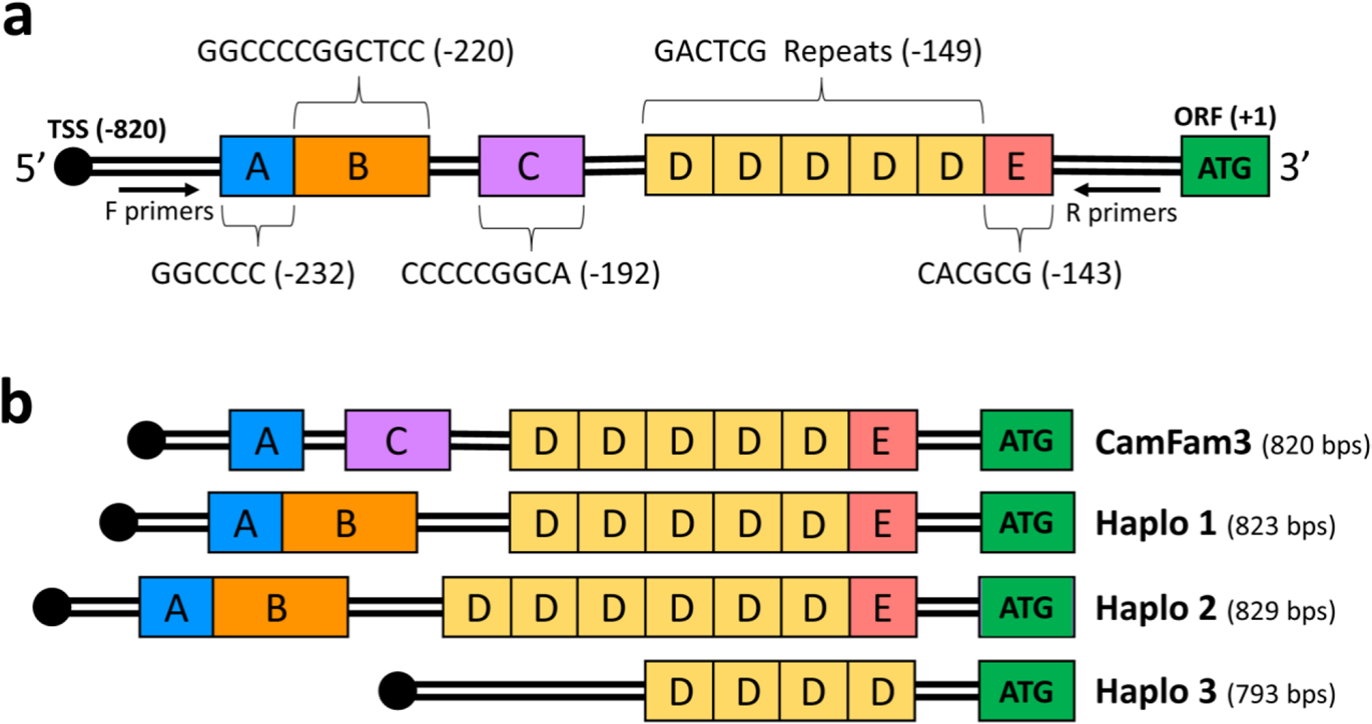
*ERBB4* 5′UTR region. **a** Schematic overview of the canine *ERBB4* 5′UTR region. Approximate location of identified indels (A, B, C, D, E) and primers used for sequencing and fragment analysis are shown. Indel sequences are displayed and positions relative to the ATG start codon (+ 1) are shown in parenthesis and based on hypothetical haplotype for which all indels are present. The TSS position is based on Uppsala University GSD1.0 gene annotations as mapped on CamFam3 [43]. **b** Schematic overview of reference and the 3 × 5′UTR haplotypes identified in GRs. 5′UTR length in parenthesis. TSS, transcription start site; ORF, open reading frame

A total of 58 GR samples representing the top associated SNP genotypes (A/A *n* = 17, A/G *n* = 17, and G/G *n* = 25) were used to further evaluate the region of interest (chr37:20,134,256–20,135,092) with Sanger Sequencing. Three haplotypes were identified within the GR *ERBB4* 5′UTR (chr37:20,134,427–20,135,247; GSD1.0 gene annotations) [44], which were distinct from the boxer breed reference genome (Canfam3.1) (Fig. 4). Haplotypes 1 and 2 differed by 6 bps, with haplotype 2 containing one additional 6-mer repeat (Fig. 4(b)). Haplotype 3 was significantly shorter than haplotype 1, with 1 fewer 6-mer repeat and 3 other upstream deletions (Fig. 4(b)), ultimately resulting in 30 bp shorter 5′UTR. The sequence alterations in the 5′UTR were all within 236 bp of the ATG of *ERBB4.*

### Development of fragment analysis for haplotyping

Because the overall length of the three haplotypes segregated in the GR, fragment analysis was used to determine the *ERBB4* 5′UTR haplotype of 381 GRs. Primers flanking the variants within the 5′UTR produced amplicons 339, 345, and 309 bps long for haplotypes 1, 2, and 3, respectively and were distinguished by the analysis software and directly correlated with the haplotypes identified using sanger sequencing.

### Association of 5′UTR variants with longevity in VMTH non-VMTH golden retrievers

**Fig. 5:**
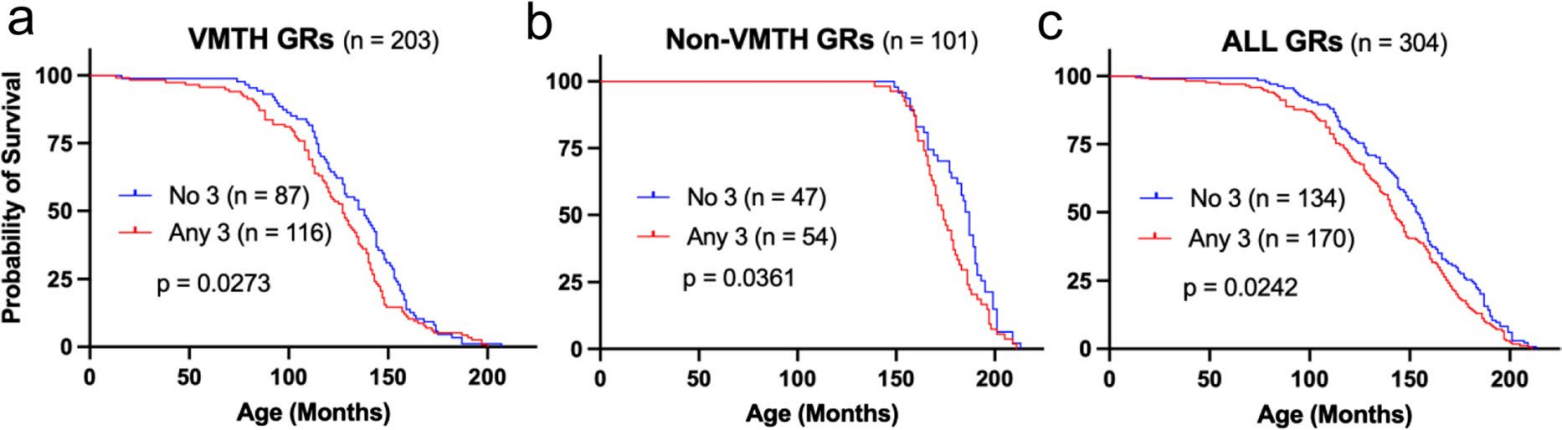
Carrying at least one copy haplotype 3 (Any 3) is associated with shortened lifespan in Golden Retriever (GR) populations. Kaplan–Meier survival curves for **a** GR patients of the Veterinary Medical Teaching Hospital (VMTH), **b** GR patients > 12 years of age submitted from owners outside of the VMTH (non-VMTH), and **c** all GR patients combined (ALL). Significant differences determined by Gehan-Breslow-Wilcoxon test

**Fig. 6:**
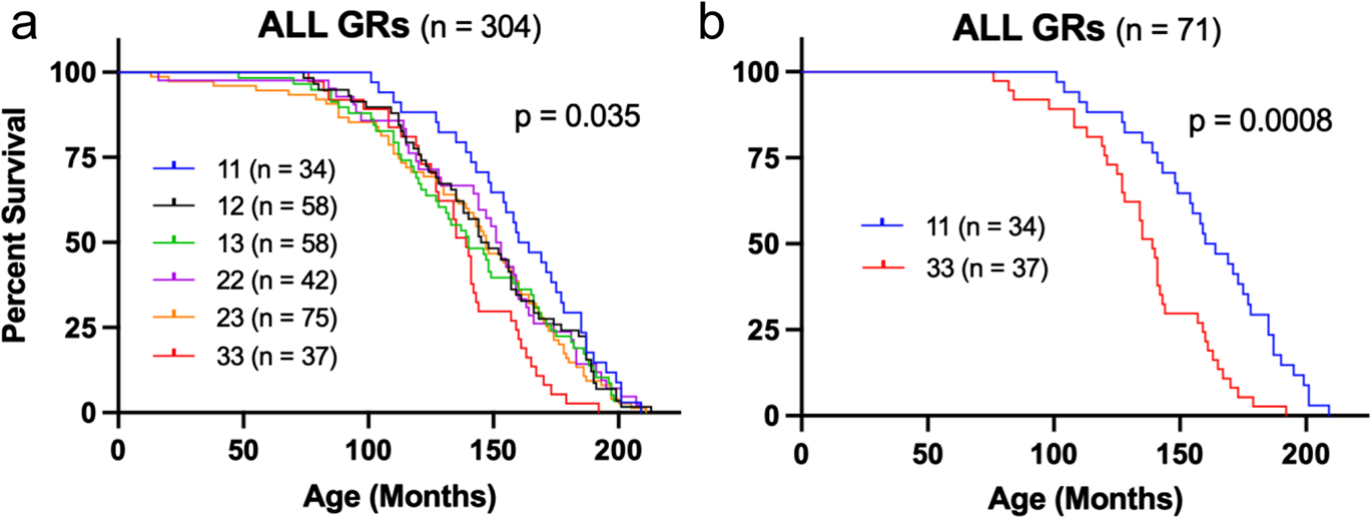
Survival of Golden Retrievers is associated with 5′UTR genotypes of *ERBB4*. Kaplan–Meier survival curves demonstrating **a** overall differences in GR lifespan associated with genotype (*p* = 0.035, Log-rank test), and **b** differences between GRs homozygous for haplotype 1 (11) and haplotype 3 (33), which have the longest (13.5 years) and shortest (11.8 years) survivals, respectively (*p* = 0.0008, Gehan-Breslow-Wilcoxon test)

Fragment analysis was initially performed on 381 GR dogs, but date of death could only be determined in 304 dogs (203 VMTH GR dogs and 101 non-VMTH GR dogs; demographics are presented in supplemental Table 2). Survival analysis performed on 203 VMTH GR patients determined that the presence of at least one copy of haplotype 3 was associated with significantly shorter lifespan (Fig. 5a) (*p* = 0.027). To validate this finding in a different population of non-referral GR dogs, the same analysis was performed on 101 GR samples submitted from outside of the UC Davis VMTH by owners of GRs over the age of 12. Within this second distinct population of GR dogs, having at least one copy of haplotype 3 was also significantly associated with reduced survival (Fig. 5b) (*p* = 0.036). When all dogs were analyzed together (*n* = 304), having at least one copy of haplotype 3 remained significantly associated with reduced survival in GRs (Fig. 5c) (*p* = 0.024). Furthermore, when all 304 dogs were analyzed by genotype, survival was significantly different between GRs based on 5′UTR genotypes, and GRs homozygous for haplotype 3 or haplotype 1 had the shortest and longest survivals, respectively (Fig. 6).

### Differential impacts of 5′UTR variants in male and female golden retrievers

**Fig. 7:**
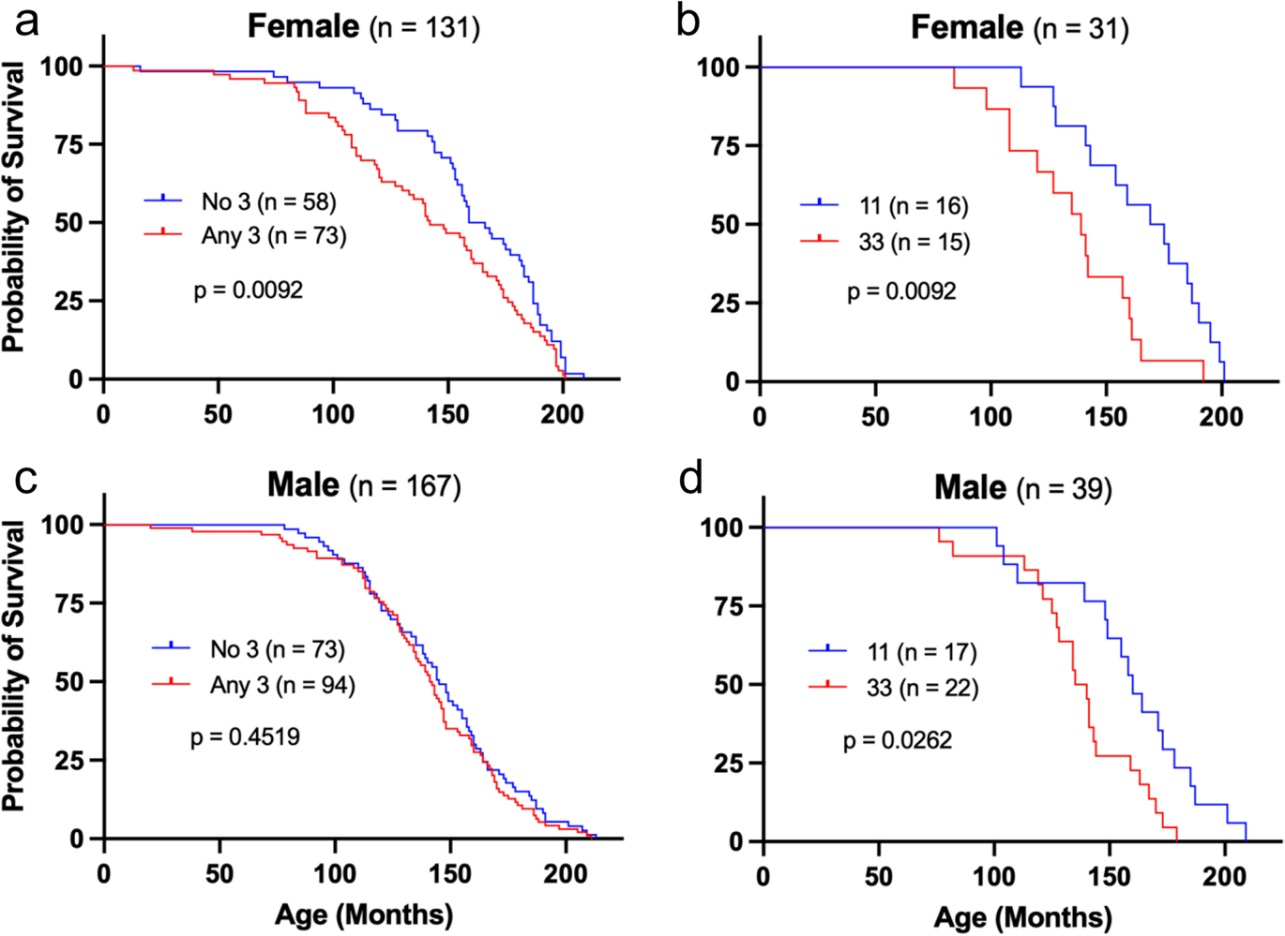
Haplotype 3–associated shortened lifespans based on GR sex. Kaplan–Meier survival curves for **a** female GR dogs with or without at least a single copy of haplotype 3, **b** female GR dogs homozygous for haplotype 1 (11) or haplotype 3 (33), **c** male GR dogs with or without at least a single copy of haplotype 3, and **d** male GR dogs homozygous for haplotype 1 (11) or haplotype 3 (33). Significant differences determined by Gehan-Breslow-Wilcoxon test

Because expression levels of *ERBB4* in canine reproductive tissues can be altered during hormonal estrus [45], and because ERBB4 is known to serve as a coactivator of the estrogen receptor [46–49], impacts of longevity associated with haplotype 3 were further analyzed by sex. Interestingly, differences in lifespan for GRs having at least one copy of haplotype 3 was seen in female dogs (*p* = 0.009, Fig. 7a) but not in male dogs (*p* = 0.452, Fig. 7c), whereas both female and male dogs homozygous for haplotype 3 demonstrated reduced survival compared with those homozygous for haplotype 1 (*p* = 0.009, Fig. 7b and p = 0.026, Fig. 7d, respectively).

### Haplotype frequency in GR population correlates with age

**Fig. 8:**
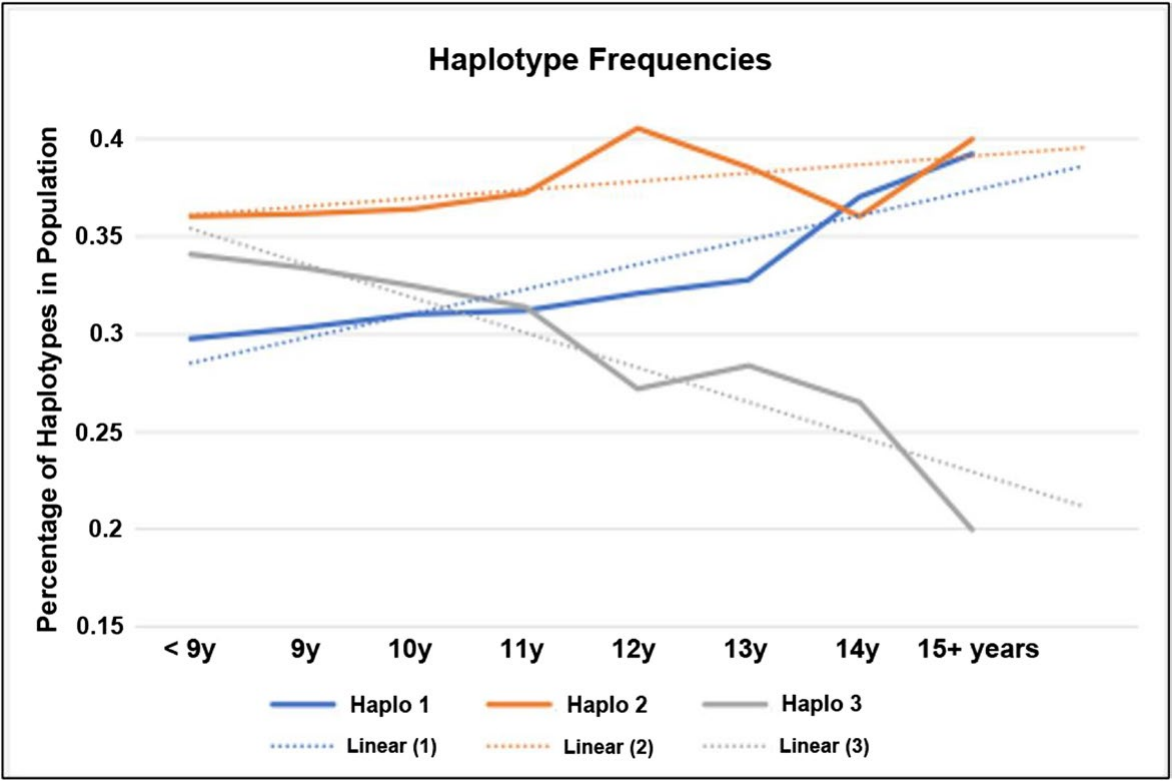
Haplotype frequencies by age. The frequency of haplotype 3 is lowest in aged GR dogs, while the frequency of haplotype 1 becomes more prevalent in older populations of GR dogs. Haplotypes from 381 GRs were determined by fragment analysis

Analysis of haplotype frequencies in 381 GR dogs as a function of age found an inverse relationship in frequency where haplotype 3 was significantly reduced with age in GR dogs, whereas the frequency of haplotype 1 increased in aged GRs (Fig. 8).

## Discussion

We set out to perform GWAS to investigate potential genes associated with longevity in GRs using whole blood DNA samples. Initially, long-lived GRs were defined as alive at 14 years and were compared with GRs who died before reaching 12 years of age. GWAS identified a SNP that reached genome-wide significance and localized to the *ERBB4* gene on chromosome 37. Fine region mapping identified 3 distinct haplotypes within the *ERBB4* 5′UTR, with the presence of one haplotype (haplotype 3) being associated with death prior to 12 years of age. Analysis of the 5′UTR haplotypes of 203 UC Davis VMTH GR patients confirmed that haplotype 3 was significantly associated with shorter lifespan, which was validated in a separate population of 101 non-VMTH GR dogs. When all dogs were analyzed together (*n* = 304), GRs homozygous for haplotype 3 had the shortest lifespan. Allele frequencies in GR dogs as a function of age also determined that the allele frequency of haplotype 3 was reduced in aged GR dogs. Finally, sex-specific analyses showed that the lifespan difference associated with possessing at least one copy of haplotype 3 was only seen in females, whereas longevity was impacted in dogs of either sex when homozygous for haplotype 3.

Despite multiple studies reporting on the average lifespan of GR dogs, defining the “long-lived” GR phenotype is not entirely clear. The median age of death in GR dogs seen at veterinary medical teaching hospitals has been shown to vary dramatically when compared to dogs seen at primary veterinary practices [7, 19, 50–52]. While this can likely be attributed to case selection bias present in populations referred to specialty centers, it does highlight the fact that the median age of death in GR within primary veterinary practices is 12.5–14 years (in contrast to 6.6–9 years in referral hospitals). However, the largest study reporting lifespan in over 9000 GR from k9data reported a median lifespan below 12.5 years of age [7]. Taken together, these studies indicate that evaluation of longevity in GRs would ideally be designed to include GR populations from both referral and primary veterinary practices, which we attempted to do by recruiting DNA samples from long-lived GRs from outside of our referral population. This data also indicates that studies focused on extreme longevity in GR should focus on dogs over the age of 14 years. Previously published GWAS studies interrogating cancer in GR were designed to identify SNPs associated with specific cancer diagnoses when compared to healthy controls over 7 or 10 years of age [31, 32], and thus were not designed to interrogate genes associated with longevity in this breed.

Our initial GWAS identified a significant association to a region of chromosome 37 which contained the *ERBB4* gene which ultimately led us to identify and focus on the 5′UTR variants. The association to *ERBB4* was certainly intriguing since GRs are predisposed to cancer and *ERBB4* is a member of the EGFR family of oncogenes. *ERBB4* is also the only member of the EGFR family that can function as either an oncogene or a tumor suppressor gene [53]. This opposing role in cancer is due to alternative functions and signaling through juxtamembrane and cytoplasmic variants, most notably JM-A, JM-B, Cyt-1, and Cyt-2 which exhibit isoform-specific roles in development and carcinogenesis [54]. Upon activation, ERBB4 forms homodimers or can heterodimerize with HER1, HER2, or HER3, and the biological effects of ERBB4 activation can be dependent on the binding ligand and the repertoire of co-expressed HER family members. Constitutive *ERBB4* tissue expression is variable but is known to be altered in human cancers including carcinomas, gliomas, and sarcomas [55–57]. GWAS studies have also associated *ERBB4* variants with polycystic ovary disease and human cancers [58–64]. ERBB4 is a co-activator of the estrogen receptor and coregulates estrogen-stimulated genes including progesterone receptor expression. Further, it has been shown to function in a growth-promoting autocrine ERBB4/ER signaling feedback loop in human breast cancer cells [46–49]. While not extensively characterized in the dog, *ERBB4* expression has been documented in female reproductive tissues and was reported to be altered during estrus cycles [45].

In addition to a well-established but varying role in tissue differentiation and human cancer, *ERBB4* is further intriguing because it has been indirectly implicated in aging and longevity. A recent study identified ERBB4 signal transduction as an overlapping pathways associated with human aging and Alzheimer’s disease [65]. ChIP-seq experiments have also shown that APOE is a molecular target of ERBB4 [66]. Furthermore, a ligand for ERBB4, neuregulin 1, was identified as a key determinant of longevity in long-lived rodent species such as the naked mole rat [67, 68].

The main finding that 5'UTR variants in *ERBB4* may be associated with lifespan in GRs needs to be interpreted with caution. While a significant association was found in two separate populations of GR dogs (referral VMTH and non-VMTH populations), several limitations exist, and additional validation studies are needed before these data should be applied clinically or in breeding programs. First, the GWAS included a very small number (*n* = 58) of GR dogs and although it reached Bonferroni significance, population stratification was noted; however, the MDS plot did not show obvious outliers. In addition, while there was no indication in the MDS plot that highly related individuals were present in our dataset, the lack of full pedigrees on GRs made it impossible to completely rule out related individuals could have been included within our population of dogs. When GEMMA was used to reduce population stratification, the region of interest on Chr37 dropped slightly under Bonferroni significance but was still highly associated with longevity (supplemental Fig. 1). Nevertheless, the GWAS led to identification of distinct 5′UTR haplotypes that segregated and were ultimately associated with longevity when expanded to include over 300 GRs from referral and non-referral GR populations.

The largely retrospective nature of this study design also meant that it was impossible to obtain definitive disease diagnoses, causes of death, or accurate histories of hormonal exposure including verification of spay neuter status and timing for all patients. Lack of known hormonal exposure in this population of GRs may be a particularly noteworthy deficiency since only females appeared to be negatively impacted from having a single copy of the 5′UTR haplotype 3. Medical records for the dogs in the VMTH population included spay and neuter status but the timing of sterilization was unknown. Therefore, it was impossible to determine the actual lifetime hormonal exposure in this retrospective population. Similarly, the non-VMTH dog owners only provided the dog’s sex and not the spay or neuter status. Due to the retrospective nature of this study, additional clinical detail was incomplete and thus other covariates possibly associated with lifespan could not be assessed such as body condition score, temperament, preventative care, diet, exercise, and environmental exposures. Prospective studies, such as the ongoing Morris Animal Foundation Golden Retriever Lifetime Study, would be ideally suited to investigate these potential covariates and for validation of our findings. Finally, the use of the age of death in client-owned dogs as a proxy for longevity also has substantial limitations since death can be impacted by the common practice of euthanasia and theoretically include death from accidental trauma or infectious disease. Several studies, however, have documented that accidental trauma or infectious disease is uncommon in mature client-owned dogs [19, 20]. Nevertheless, the lack of a definitive cause of death for all patients represents a weakness of this study.

Many of the VMTH dogs in our study had definitive diagnoses of malignant cancer prior to their death. For these dogs, even in the absence of necropsy, it would be reasonable to assume that cancer was the primary cause of death or their owner’s decision to euthanize. In long-lived GRs, where owners may be less inclined to pursue invasive and expensive diagnostics and similarly less interested in a definitive diagnosis by necropsy, attributing cancer to the cause of death becomes more subjective. For example, several of the long-lived GRs in our GWAS study were euthanized after developing neurologic signs. While a cancer diagnosis may be suspected, in the absence of necropsy or advanced diagnostics including advanced imaging or biopsy, neurologic signs secondary to metabolic, infectious/inflammatory, or degenerative processes cannot be ruled out. Conversely, some owners reported reduced quality of life attributed to aging or degenerative processes prior to death, but without diagnostics or necropsy, cancer and other diseases cannot be ruled out. Although we included cancer diagnoses when known, this data should be considered incomplete and interpreted with caution.

Ongoing studies in our group are currently focused on potential implications of the 5′UTR haplotypes to determine if variants in this region could play a functional role in altered transcription, translation, RNA stability, or splicing; leading to altered levels of *ERBB4* or altered expression of variants with oncogenic or tumor suppressive functions across breeds. Additional studies are now being directed towards evaluating *ERBB4* variants in other breeds, within canine tumors, and other species including mouse and human genomes.

### Supplementary Information

Below is the link to the electronic supplementary material.

Supplementary file1 (DOCX 35 KB)

## Data Availability

Genotype data has been submitted and is publicly available through Dryad (https://doi.org/10.5061/dryad.3j9kd51r2). Whole-genome sequence data was deposited in SRA (PRJNA961733: SRR24300331- SRR24300335, SRR24300345, SRR24300356, and SRR24300357).
