# Supplementary material for: A variant in the 5′UTR of ERBB4 is associated with lifespan in Golden Retrievers
Source: GeroScience. 2023 Oct 19;46(3):2849–62. doi: 10.1007/s11357-023-00968-2 (PMC11009206; doi:10.1007/s11357-023-00968-2)
Supplement: Supplementary file 1 — Supplementary file1 (DOCX 35 KB) [file 11357_2023_968_MOESM1_ESM.docx]

GR Longevity – Supplemental

|  | **Death**  **< 12 y** | **Death**  **≥** **14 y** |
| --- | --- | --- |
| Number of GR Dogs | 29 | 29 |
| Median age of  Death (range) | 102 months  (169-213) | 189 months  (13-143) |
| Median Weight  (range) | 33.2 kg  (24-45.5; n=29) | 33.4 kg  (28-37.4; n=7) |
| **Sex** | | |
| Male | 18 | 14 |
| Female | 11 | 14 |
| Not listed | 0 | 1 |
| **Cancer Diagnosis** | | |
| Confirmed | 27 | 5 |
| Suspected | 2 | 4 |
| None reported | 0 | 8 |
| Unknown | 0 | 12 |

**Supplemental Table 1. Demographics of the 58 GRs used in GWAS.** GRs were grouped into 2 categories, those who died younger than 12 years (death < 12 y) and those who lived to 14 years or older (death ≥ 14 y). Cancer diagnosis indicated here are reported at any point in the patient’s lifetime and does not necessary indicate cancer as the definitive cause of death.  **“**Confirmed” cancer diagnosis was determined by cytology or histopathology (VMTH patients) or provided in the owner history (non-VMTH).  “Suspected” cases include patients with symptoms or imaging consistent with cancer, but not confirmed with cytology or histopathology (VMTH and non-VMTH). “None reported” include patients in which cancer was not suspected during their lifetime (based on owner history).  Patients for which no data was available to indicate or rule out cancer were marked “Unknown”.

|  | | **VMTH** | **Outside** |
| --- | --- | --- | --- |
| Number of GR Dogs | | 203 | 101 |
| Median age of Death  (range) | | 130 months  (13-207) | 179 months  (139-213) |
| **Sex** | | | |
| **Male** | Intact | 45 | 0 |
|  | Castrated | 75 | 5 |
|  | Unknown | 0 | 42 |
| **Female** | Intact | 9 | 0 |
|  | Spayed | 70 | 6 |
|  | Unknown | 0 | 46 |
| Sex Not listed | | 4 | 2 |
| **Cancer Diagnosis** | | | |
| Confirmed | | 146 | 21 |
| Suspected | | 27 | 13 |
| None reported | | 22 | 36 |
| Unknown | | 8 | 31 |

**Supplemental Table 2. Demographics of the 304 GRs with known dates of death used in survival analysis.** Cancer diagnosis indicated here are diagnosis reported at any point in the patient’s lifetime and does not necessary indicate cancer as the definitive cause of death.  **“**Confirmed” cancer diagnosis was determined by cytology or histopathology (VMTH patients) or provided in the owner history (non-VMTH).  “Suspected” cases include patients with symptoms or imaging consistent with cancer, but not confirmed with cytology or histopathology (VMTH and non-VMTH). “None reported” include patients in which cancer was not suspected during their lifetime (based on owner history).  Patients for which no data was available to indicate or rule out cancer were marked “Unknown”.


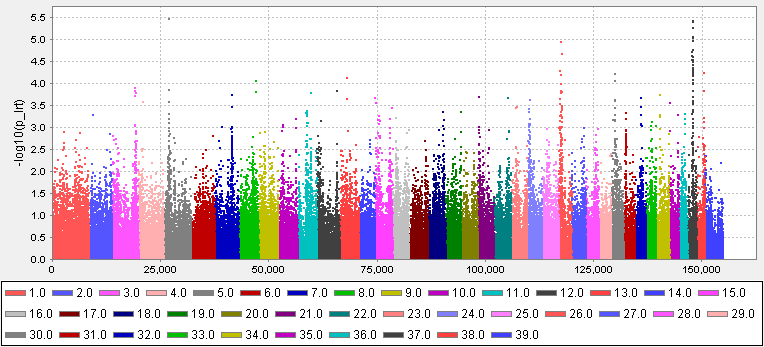


**Supplemental Fig 1.** GEMMA corrected Manhattan plot of longevity association in Golden retrievers. The highest associated SNVs are found in chromosomes 5, 13, and 37. Although GEMMA decreased the SNV on chromosome 37 below Bonferroni significance (p-value of 3.56 x10^-7^), it was still highly associated with longevity.
